# TRANSCENDING ‘INTERDISCIPLINARY PARADOX’: TOWARDS AN AUTONOMOUS BODY OF KNOWLEDGE THROUGH TRANSDISCIPLINARITY

**DOI:** 10.1093/geroni/igae098.3910

**Published:** 2024-12-31

**Authors:** Shiyu Chen, Shandong Zhou

**Affiliations:** Hunan Women’s University, Changsha, Hunan, China (People’s Republic); Xiangtan University, Changsha, Hunan, China (People’s Republic)

## Abstract

Autonomous body of knowledge (BoK) in gerontology is not only an extrinsic requirement to address the challenges of an aging society, but also an intrinsic need for the discipline to achieve its independency. The establishment of such a BoK requires an introspective reflection. This poster presents a review of the progress on the establishment of gerontology’s autonomous BoK, and explores the evolvement of the discipline following the “multidisciplinary-interdisciplinary-transdisciplinary” continuum. The study finds a ‘interdisciplinary paradox’ in current gerontological BoK, which manifests as: programmatic discourse on interdisciplinarity in gerontology continues to proliferate and disseminate, yet disciplinary and sub-disciplinary differentiation and specialization are ever-increasing. Moreover, there remains structural barriers in academia supporting interdisciplinary research in gerontology. To resolve this paradox and reinforce the establishment of gerontological autonomous BoK, a transdisciplinary research model (ISOE model) consisting of 3 phases could be adopted. The 3 phases in the model are as following: a. Breaking down barriers between organizations, interacting with diverse stakeholders and framing the real-world problem collectedly b. Integrating research methodologies and perspectives, and collaborating on producing solution-orientated knowledge and transferable results; c. Translating and applying research results to real world practice. In conclusion, transdisciplinary method offers a promising pathway to establish an autonomous BoK in gerontology.

